# FOCAD Gene Defect Resulting in Rapidly Progressing Neonatal Liver Cirrhosis Requiring Transplant

**DOI:** 10.7759/cureus.93764

**Published:** 2025-10-03

**Authors:** Saarah Raja, Luana Ayres Da Silva, Mithuna Urs

**Affiliations:** 1 Paediatrics and Neonatology, Tunbridge Wells Hospital, Kent, GBR

**Keywords:** focad gene defect, focadhesin, liver cirrhosis, liver transplantation, neonatal

## Abstract

In children, cirrhosis is rare and most commonly associated with biliary atresia and other metabolic or genetic conditions. We report a rare case of a preterm infant who developed severe liver cirrhosis in the first few months of life, requiring liver transplant at three months of age. She presented with persistent hypoalbuminaemia, peripheral oedema, ascites, metabolic bone disease with fractures, coagulopathies, and anaemia. She subsequently developed multiorgan failure and disseminated intravascular coagulation (DIC). Extensive investigation found FOCAD gene defect, compound heterozygous for c.4435del p.Lys1475Asnfs* and exon 6-7 deletion. In conclusion, we hope to contribute to the awareness and growing understanding of FOCAD deficiency in children. This case highlights the importance of early diagnosis and consideration of screening for FOCAD deficiency in cases where the cause for cirrhosis is unknown. More research will be needed to better elucidate the full spectrum of the condition, as well as to establish potential prognostic markers and therapeutic targets.

## Introduction

FOCAD is a gene identified as being essential for liver health. It encodes for the protein focadhesin, whose specific function and structure remain undetermined [1]. Levels of FOCAD are reported to be highest in the brain [2], and loss of heterozygosity has been associated with the development of glioma and colorectal cancer [2-4]. A study by Traspas et al. identified 14 children with severe neonatal liver cirrhosis [1]. All participants were found to have germline recessive mutations in FOCAD, not previously associated with liver function. The study has shown that "loss of FOCAD, operating via the SKI messenger RNA surveillance pathway, causes a paediatric syndrome with liver cirrhosis" [1]. This pathway is associated with hepatocyte health, and FOCAD defect results in reduced levels of RNA helicase SKIC2 and its cofactor SKIC3, leading to inflammation, hepatocyte injury, reduced albumin, and cirrhosis [1].

A study by Devarbhavi et al., looking at global burden, has found that liver disease accounts for around two million deaths annually, equating to one in 25 deaths [5]. These deaths are largely due to complications of cirrhosis and hepatocellular carcinoma, as opposed to acute causes. Cirrhosis may be defined as an irreversible condition, characterised by hepatic fibrosis and nodule formation [6]. In the initial stages, factors such as developmental abnormalities, infections, and metabolic and genetic disorders may damage the liver. Initially, the liver forms scar tissue known as fibrosis, and its structure remains intact. Over time, this repetitive injury can lead to extensive fibrosis with loss of structure, resulting in cirrhosis [6]. Although neonatal liver cirrhosis is reported to be rare, rates of morbidity and mortality are significant. Individuals may present with faltering growth and can display signs of malnutrition. Jaundice, ascites, and a distended abdomen with liver and/or spleen enlargement can also be present [7].

We report a case of neonatal FOCAD gene defect resulting in cirrhosis and requiring a cadaveric liver transplant. We aim to raise awareness of this rare condition in the hope of improving outcomes and quality of life for these patients.

## Case presentation

Our case reports a case of a singleton baby girl, born at 31+1 weeks, in good condition, weighing 1000 g. The baby received inflation breaths soon after birth and then continuous positive airway pressure (CPAP). Appearance, Pulse, Grimace, Activity, and Respiration (APGAR) scores were recorded as 9, 10, and 10 at one, five, and 10 minutes, respectively. She was delivered via emergency C-section due to abnormal cardiotocography (CTG), intrauterine growth restriction (IUGR), and breech presentation. The parents were non-consanguineous and Caucasian (white: other). The mother was aged 33 at delivery and had a miscarriage (at eight weeks of gestation) one year previously, and this was a pregnancy following in vitro fertilisation (IVF). IUGR and preeclampsia were identified during the pregnancy, and the mother was taking labetalol and nifedipine for hypertension. Magnesium sulphate was given for foetal neuroprotection, and betamethasone was also given for foetal lung maturation.

The baby was admitted to a level 2 neonatal unit due to prematurity. She remained on CPAP for one day, followed by two days on high-flow oxygen via Vapotherm. She subsequently self-ventilated in air. As per unit protocol, she received 48 hours of intravenous antibiotics for suspected sepsis and had negative C-reactive proteins (CRPs) and blood cultures. She was also treated with phototherapy for jaundice. The baby was also noted to have hypoalbuminaemia since birth, with the highest value being 28 g/L, but there were no other concerns at that point.

Around 14 days of life, she had bilious aspirates overnight. On examination, her abdomen was soft but distended. A feed was held at this time and then restarted. At 16 days of life, she had three hypoglycaemic episodes, with a lowest recorded blood sugar (BM) of 1.9 mmol/L. A hypoglycaemia screen was done, revealing hyperinsulinaemia, and plasma amino acids showed persistently raised tyrosine levels. After discussion with a specialist endocrine team, it was agreed for the baby to have high-energy formula (Maxijul, a carbohydrate energy supplement) via nasogastric (NG) tube/bottles and regular BM monitoring. Regular diazoxide was also started due to the hyperinsulinaemia. Additionally, on day 16 of life, a capillary haemangioma was observed on the left upper arm.

Around eight weeks of life, she had worsening peripheral oedema (of the legs and groin with extension into the abdominal wall). Around this point, it was also noted that the baby was not responding to the regular diazoxide as expected. The parents were informed of this and were notified that she may need further testing for metabolic conditions. The baby was also started on diuretics due to the peripheral oedema and persistent hypokalaemia. Abdominal ultrasound, around nine weeks of life, confirmed new ascites with normal portal system Dopplers and other intra-abdominal organs, but an inguinal and umbilical hernia were reported. The ascites at this time was thought to be related to the low hemoglobin, albumin, and osmotic drive. Increasing abdominal girth (38 cm recorded at 10 weeks of age) was also noted.

The infant had ongoing hypoalbuminaemia (for which she received two albumin infusions), with repeat coagulation profile showing raised prothrombin time (PT) and activated partial thromboplastin time (APTT). Due to the deranged coagulation, she received vitamin K and fresh frozen plasma (FFP) at 11 weeks of life. At this point, the baby's various medical issues had been discussed with multiple endocrine teams and local tertiary units, with a lack of a unifying diagnosis.

Additionally, she was found within the first few months of life to have a patent ductus arteriosus, metabolic bone disease with alkaline phosphatase (ALP) >1000 (initially managed with phosphate, vitamin D, and calcium supplementation). X-rays confirmed fracture of the left wrist and left neck of the femur, and she was later noted to have fractures of the right upper limb and ribs. She was also found to have clotting derangements, initially treated as disseminated intravascular coagulation (DIC). She also had multiple episodes of anaemia with a total of five packed red blood cell (RBC) transfusions within the first few months of life.

**Figure 1:**
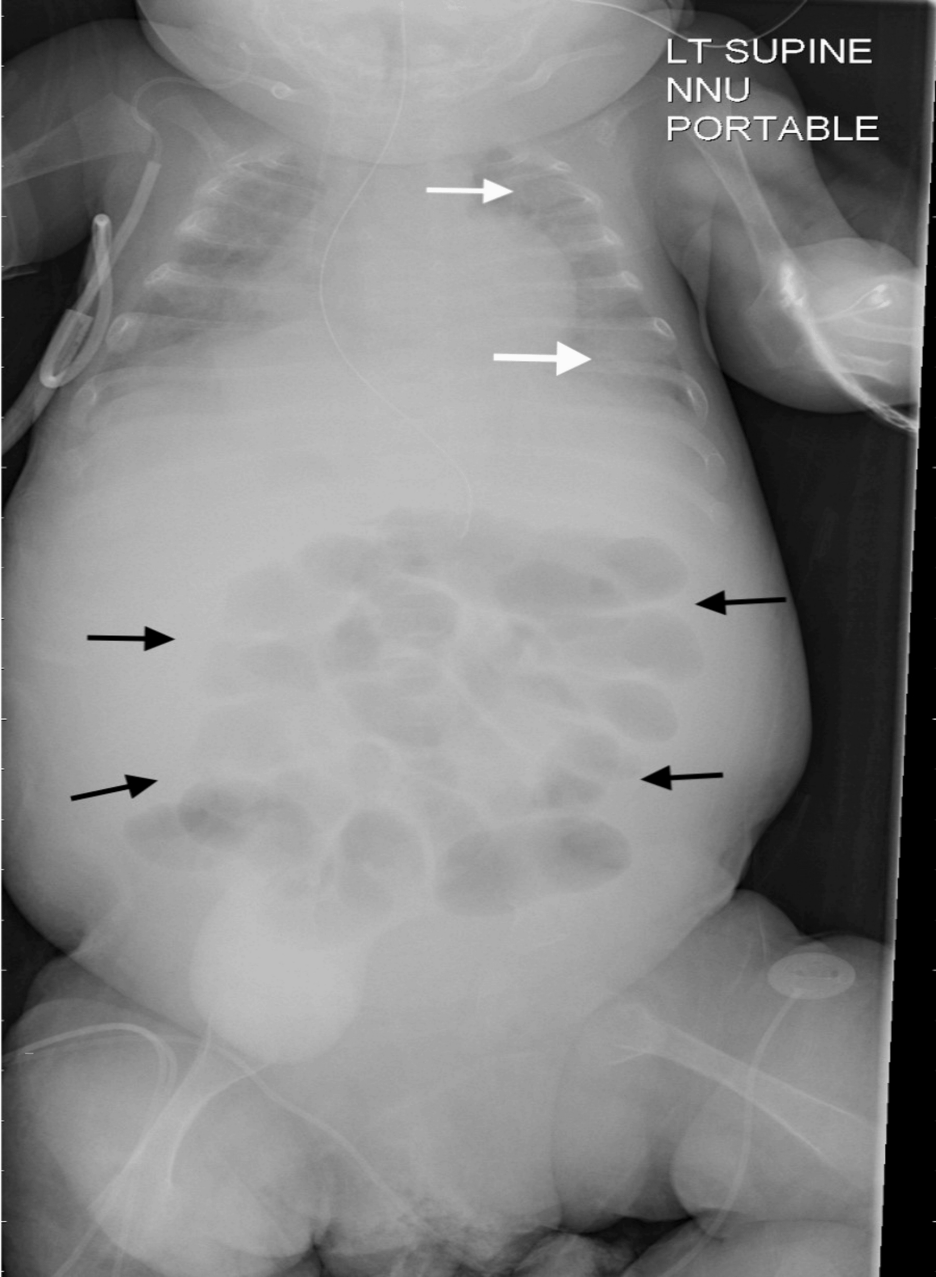
Chest and abdominal X-ray Chest and abdominal X-ray taken on day 81 of life, following respiratory deterioration. There is left upper and lower lobe consolidation (white arrows), as well as small lung volume bilaterally. Due to ascites, there is poor definition of the liver, and bowel loops are displaced to the centre of the abdomen (black arrows).

At the age of two months and three weeks (day 81 of life), the baby developed respiratory deterioration secondary to ascites, with left lobe consolidation and right upper lobe collapse, as shown in Figure 1. She subsequently developed multiorgan failure and DIC, which required transfusion with multiple blood products.

She was transferred from the neonatal unit to a paediatric intensive care unit (PICU), at a tertiary centre with liver specialists, on day 84 of life (two months and three weeks old). This was in view of progressive respiratory decompensation requiring intubation and mechanical ventilation.

**Figure 2:**
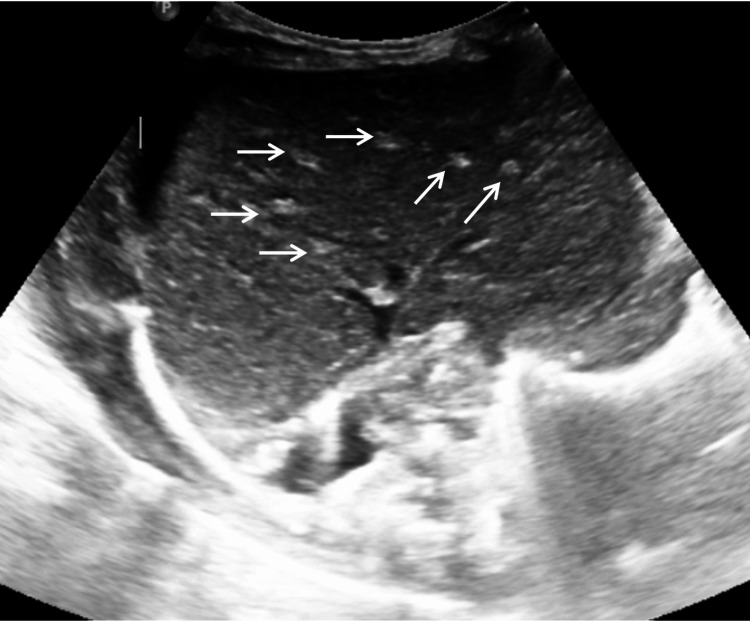
Oedematous liver with echogenic portal triads (shown by white arrows) giving the liver a starry-sky appearance These changes contributed to rapid cirrhosis and portal hypertension, requiring liver transplant at three months of age.

Extensive investigation found that she had FOCAD gene defect, compound heterozygous for c.4435del p.Lys1475Asnfs* and exon 6-7 deletion. An ultrasound of her liver is shown below in Figure 2. She developed rapid cirrhosis and portal hypertension and required a liver transplant at three months of age. She received a left lateral segment, single artery, single duct Roux loop from donation after brainstem death (DBD). Due to the status of the donor being *Streptococcus pneumoniae* type A positive and/or the presence of bacterial meningitis, she was also treated with ceftriaxone and metronidazole. At this point, the abdomen was temporarily closed with a silicone mesh. One month later, she was taken to surgery again, and a smaller mesh was used to close the abdomen. Three weeks after this, at five months and one week of age, the baby received a fascia donation to help with the closure of her abdomen. The baby had a prolonged postoperative stay in the PICU/high-dependency unit (HDU), requiring increased respiratory support. She was stepped down to the ward at six months of life and discharged home at seven months of age.

She is currently followed up by various teams, including general paediatrics, paediatric hepatology, paediatric endocrinology, and immunology. A few months after the transplant, she had persistently raised liver enzymes due to mild rejection, but this has now improved, and there is no current concern with her liver function. She is clinically not jaundiced, liver function tests (LFTs) are in a good range, immunology tests are normal, and she is neurodevelopmentally well, achieving milestones. The haemangioma on the left arm is also noted to be involuting well. She remains on the following medications: tacrolimus twice daily, azathioprine once daily, prednisolone once daily, and protein-bound magnesium tablets.

A summary of her blood results can be seen in Table 1 below.

**Table 1:** Main blood test results Column A represents her first blood results on the day of birth. We can observe persistent hypoalbuminaemia from A to G (prior to liver transplantation) and progressively rising ALP and high GGT and AST levels. Column H represents her results three months after liver transplant. Finally in column I, we have her most recent blood results, at three years of age. Deranged values are in bold font. ALT: alanine aminotransferase; ALP: alkaline phosphatase; GGT: gamma-glutamyl transferase; AST: aspartate aminotransferase; Hb: hemoglobin; PT: prothrombin time; APTT: activated partial thromboplastin time; INR: international normalized ratio

Parameters (including reference ranges)	A	B	C	D	E	F	G	H	I
Albumin (30-45 g/L)	27	21	20	19	24	25	23	36	40
ALT (<35 U/L)	7	7	16	20	18	25	21	73	33
ALP (122-469 U/L)	331	602	603	935	1664	2141	809	616	265
Bilirubin (<290 µmol/L)	81	21	16	29	63	106	167	<3	4
GGT (6-42 IU/L)	-	-	-	-	-	143	82	193	10
AST (<35 U/L)	-	-	-	-	-	48	39	147	-
Hb (115-165 g/L)	149	85	73	67	102	107	82	117	135
Platelet count (200-500×10^9^/L)	177	148	196	183	264	201	50	160	211
PT (11-15 seconds)	-	-	-	-	-	20.2	-	15.6	-
APTT (23-37 seconds)	-	-	-	-	-	42.9	54.3	42.7	-
Fibrinogen (1.5-5 g/L)	-	-	-	-	-	1.21	3.06	4.36	-
INR (0.9-1.3)	-	-	-	-	-	2.3	2.6	-	1.2

## Discussion

Causes of liver cirrhosis are well understood and established in adult patients [8]. In children, inherited causes are more commonly associated with liver disease leading to cirrhosis. Causative conditions include Wilson's disease, alpha-1-antitrypsin deficiency, primary sclerosing cholangitis, biliary atresia in infants, Alagille syndrome, mitochondrial hepatopathies, hepatitis, MDR3 deficiency, FIC1 deficiency, and glycogen storage disorders [9,10]. The current gold-standard treatment for both paediatric and adult patients with cirrhosis is liver transplantation. 

The association of FOCAD with implications in liver cirrhosis is relatively novel, and as such, the literature is limited. In terms of biological function, the gene has been associated with functions involved in focal adhesions, microtubule dynamics, cell cycle control, as well as cancer predisposition [2-4,11]. Studies by Traspas et al. analysed livers from focad-knockout zebrafish, which demonstrated defects in the SKI mRNA surveillance pathway. SKI is a multiprotein complex, composed of helicase SKIC2 and its cofactors SKIC3 and SKIC8. Its function is to associate with the cytoplasmic exosome in order to break down RNA molecules [12-14]. This study has shown FOCAD to be fundamental in maintaining proteostatic levels of SKIC2 and SKIC3 in several human cell lines as well as in an animal model of the disease. 

Our case aligns with recent literature on FOCAD deficiency causing liver cirrhosis in children. In the 2022 study mentioned earlier, Traspas et al. reported a paediatric cirrhotic disorder in 14 children who belonged to 10 unrelated families from seven countries, with variants of FOCAD deficiency. Early-onset cholestasis, nodular cirrhosis, hepatomegaly, hypoalbuminaemia, splenomegaly, jaundice, ascites, and portal hypertension were seen in the children, with acute liver decompensation often occurring in infancy. Additionally, gastrointestinal issues were also observed in the 14 children, including abdominal distension, feeding difficulties, inguinal and umbilical hernias, as well as other problems such as IUGR and metabolic (increased glycogen content) and blood disorders. In total, six of the 14 children passed away from hepatic or multiorgan failure: five of them before one year of age and one child two weeks after liver transplantation at six months of age [1]. 

Comparatively, our case expands the phenotypic spectrum of FOCAD deficiency, describing a severe, early-onset manifestation and rapid progression of liver disease, requiring liver transplant at three months of life. In contrast, our case also demonstrates how early diagnosis of the underlying condition and early intervention with liver transplant can improve the outcome, as the child is now thriving at three years of age. 

## Conclusions

We hope to contribute to the understanding of FOCAD gene deficiency and its spectrum in children. We understand that more research is needed to better elucidate the full spectrum of the condition and its different phenotypes and to establish potential prognostic markers and even new therapeutic targets. We also aim to raise awareness of the importance of early detection of liver dysfunction and spread the message that early genetic testing should be considered for infants with unexplained hypoalbuminaemia and liver failure. This will allow early intervention and improve the outcome, as seen in our reported case. Further genetic studies will also benefit parents and help clinicians involved in counselling these families. 
